# Surgery

**Published:** 1905-09-30

**Authors:** 


### SURGERY.

### FRACTURES.

**Fractures of the Sternum.**—Fractures of the sternum are by no means so rare if the pathological specimens in our museums and post-mortem experience are taken into consideration. Yet neither the older writers on fractures nor clinical experience appear to bear out the truth of this. The fact is, a fracture of the sternum at its most common site through the manubrium is extremely difficult, or almost impossible, to diagnose from a dislocation between the manubrium and gladiolus. Putting aside fractures of the sternum from direct violence which have no special significance, and also those rare cases due to violent muscular contraction, such as forcible bending of the spine backwards, we believe that by far the greatest number as Dr. G. de Tarnowsky (1) points out are due to contre coup. This author correctly states that contre-coup fractures of the sternum are usually produced by falls on the head and shoulders, the thorax being brought into a position of forcible antiflexion. The lesion in the majority of instances being situated about the junction of the manubrium and gladiolus, the latter portion of the bone is displaced behind the lower end of the upper fragment. The exact mechanism is still quite unsettled. Tarnowsky summarises the factors as follows:—1. Falls on the head and shoulders pressing the ribs forwards and upwards. 2. The clavicles may sometimes act as a lever and help to wrench the manubrial portion from the gladiolus. 3. Intrathoracic pressure at the time of the fall exerts a positive pressure upon the thoracic wall. 4. The second costal cartilages act as a wedge, tending to separate the manubrial portion from the gladiolus. In order to effect reduction the patient is placed on his back with his head and neck hanging over the table, while the upper extremities are brought upwards above the head and rotated outwards. The spine is strongly extended by downward traction of the chin and occiput, the legs and pelvis being fixed. If the displacement is reduced by these means a plaster of Paris splint is applied to fix the chest. Operative treatment by incision and elevation of the fragments is only indicated wherepressure symptoms such as cyanosis, dyspnœa, or the formation of an abscess in the anterior mediastrium, threaten life.

**Fractures of the Cervical Vertebræ.**—The advent of the *x*-ray has revealed the fact that fractures of the cervical vertebræ are by no means uncommon. This is especially true of the second cervical vertebra in which, for example, fracture of the odontoid process has not even been suspected. Dr. O. Horwitz[2] relates the interesting case of a man who had been injured by the fall of a heavy weight, which struck him on the shoulder and knocked him backwards striking the back of his neck on a "roll of goods." On recovering consciousness he was able to ride eight miles in a trolley-car, and then walked home a distance of a quarter of a mile. Although there was some stiffness of the back of the neck no displacement or irregularity of the spine or paralysis or anæthesia could be detected. Complete paralysis, however, appeared on the second day and lasted for eight days. Finally, a gradual but almost complete recovery ensued. From the hard mass which persisted over the second cervical vertebra it was clear that there had been a fracture of this bone. A second case reported by Dr. R. H. Harte[3] of fracture of the odontoid process of the axis, which gave rise to few or no symptoms is even of greater significance in as much as the fracture was only revealed by an autopsy. It occurred in the case of a brakeman who was struck by the step of a caboose, and sustained fractures of the clavicle and jaw. The man walked more than a mile to the hospital, where the fractures were attended to, but he did not complain of anything wrong with his neck. Shortly afterwards turning in bed he died instantly. There were found fractures of the bodies and spinous processes of some of the cervical vertebræ, which were quite recoverable had not there been a complete fracture of the odontoid process. Drs. F. T. Stewart and J. B. Roberts[4] have also recorded instances of fracture of the cervical vertebræ unrecognised until revealed by the *x*-ray during routine examination. In the two related by the former author there were no symptoms referable to the spine, and no callus had formed, but in the case of the latter author there was paralysis of all the extremities following a fall, and on carefully going into the history it was found that he had injured his neck six months previously, and that at that time a finger introduced into the mouth could detect a mass in the region of the fourth or fifth cervical vertebræ. The paralysis was probably due to the tearing of some of the adhesions round the old injury producing hæmorrhage or the effusion of serum. In twenty-four hours power in one arm returned, and afterwards in both legs, followed by sensation. At the end of six months there was almost perfect use in all four extremities.

**Upper Extremity.**—*Injuries of the Elbow Joint especially to the Radius.*—There are no injuries which to the advantage of the practical surgeon have received greater elucidation by the *x*-ray than those at the elbow joint. Indeed, as Mr. Poland in a recent lecture[5] has stated, our knowledge of these has so vastly increased that a more complete classification and a thorough exposition of them are demanded at the present time. It is a subject of regret that nowadays there is no complete treatise on injuries of the elbow joint, and no complete skiagraphic atlas of this articulation in its various positions in infancy, youth, and adult age to assist the surgeon in unravelling these complex injuries. Mr. Poland has attempted by numerous skiagraphs in his lecture to show the very common occurrence of two groups of injury—namely (1) to the upper end of the radius whether dislocation or fracture of the head or neck; and (2) of external oblique fracture of the humerus into the elbow joint. As regards the first group he thinks that (apart from subluxation of the radial head) dislocation is not at all an uncommon injury, and can be completely remedied by operation such as he has performed in several cases. Fracture of the neck of the radius he illustrated by skiagraphs of the elbow before and after operation taken from a gentleman aged 43, who had sustained a dislocation of both bones of the forearm backwards accompanying an injury of this description. The dislocation having been reduced at the time of the accident, the fractured head of the radius was subsequently found to be displaced forwards and inwards, lying between the brachial artery and median nerve with its humeral cup presenting forwards, beneath the skin. There were signs of pressure on the nerve such as tingling in the tips of the index, middle, and ring fingers. Removal of the displaced radial head resulted in almost perfect recovery of function of the joint. This example rightly led the lecturer to the difficult subject of rotation of the displaced and fractured portion of bone or bones—a subject which has not been sufficiently recognised in injuries of the elbow joint but which is a most important feature in the treatment of these injuries. The *x*-ray has done much to emphasise this complication of these injuries and we hope it will have the careful consideration of surgeons in the future.

[1] Annals of Surg., February 1905, p. 252. [2] Ibid., March 1905, p. 472. [3] Ibid., p. 473. [4] Ibid., p 474. [5] Clin. Jour., July 19, 1905.

